# Cellular Signaling Pathways in Medium and Large Vessel Vasculitis

**DOI:** 10.3389/fimmu.2020.587089

**Published:** 2020-09-25

**Authors:** Ryu Watanabe, Gerald J. Berry, David H. Liang, Jörg J. Goronzy, Cornelia M. Weyand

**Affiliations:** ^1^Department of Medicine, Stanford University School of Medicine, Stanford, CA, United States; ^2^Department of Pathology, Stanford University School of Medicine, Stanford, CA, United States

**Keywords:** giant cell arteritis, Takayasu arteritis, large vessel vasculitis, T cells, macrophages, NOTCH, co-stimulation, immune checkpoint

## Abstract

Autoimmune and autoinflammatory diseases of the medium and large arteries, including the aorta, cause life-threatening complications due to vessel wall destruction but also by wall remodeling, such as the formation of wall-penetrating microvessels and lumen-stenosing neointima. The two most frequent large vessel vasculitides, giant cell arteritis (GCA) and Takayasu arteritis (TAK), are HLA-associated diseases, strongly suggestive for a critical role of T cells and antigen recognition in disease pathogenesis. Recent studies have revealed a growing spectrum of effector functions through which T cells participate in the immunopathology of GCA and TAK; causing the disease-specific patterning of pathology and clinical outcome. Core pathogenic features of disease-relevant T cells rely on the interaction with endothelial cells, dendritic cells and macrophages and lead to vessel wall invasion, formation of tissue-damaging granulomatous infiltrates and induction of the name-giving multinucleated giant cells. Besides antigen, pathogenic T cells encounter danger signals in their immediate microenvironment that they translate into disease-relevant effector functions. Decisive signaling pathways, such as the AKT pathway, the NOTCH pathway, and the JAK/STAT pathway modify antigen-induced T cell activation and emerge as promising therapeutic targets to halt disease progression and, eventually, reset the immune system to reestablish the immune privilege of the arterial wall.

## Introduction

Arterial blood vessels are categorized according to their diameter into large, medium, and small vessels. Large vessels are the aorta and its major branches, medium vessels are the main visceral arteries and the arteries supplying blood to the brain, and small vessels are intraparenchymal arteries (1). Common elements are the lumen-lining endothelial cells (intimal layer) and the smooth muscle cells enabling dynamic change of diameter and resistance (medial layer). The outermost layer (adventitial layer) contains connective tissue, nerves, and vasa vasorum networks to supply nutrients and blood to the wall (2). Notably, each category of arteries is subject to different disease processes. Atherosclerotic disease, now recognized as a smoldering inflammatory process triggered by subendothelial lipid deposits that spans multiple decades of life, is a major cause of morbidity and mortality (3–5). In contrast, autoimmune inflammation of arteries is a more aggressive process, complicated by hemorrhage, vessel rupture and vessel occlusion. Vasculitides share immunopathologic features with other autoimmune diseases but have specifying immune abnormalities and clinical manifestations that are related to the life-sustaining role of arteries (1).

**FIGURE 1:**
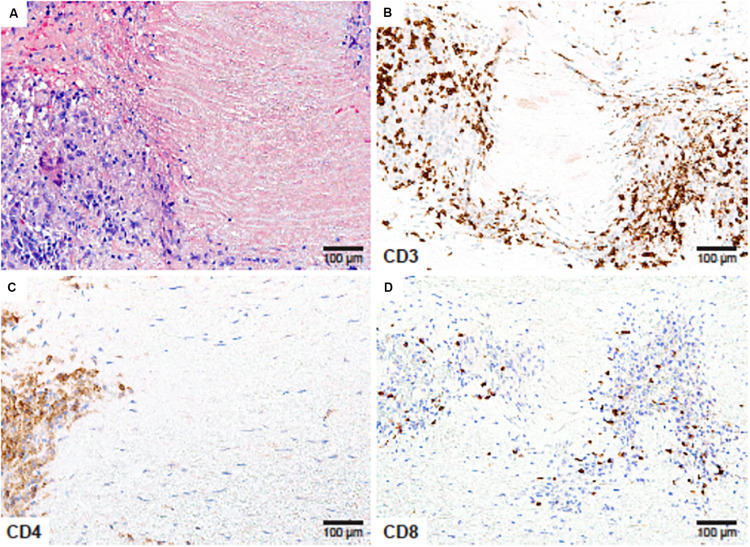
CD4^+^ and CD8^+^ T cells in Giant Cell Arteritis. Tissue sections from an ascending aortic aneurysm repair in a 66-year-old woman presenting with back pain in the mid-thoracic region. **(A)** High power magnification of the medial layer showing granulomatous inflammation surrounding depleted smooth muscle cells and necrotic elastic fibers (Hematoxylin and eosin ×200). **(B)** CD3 immunostaining showing numerous T cells within the medial infiltrates (×200). **(C)** CD4 immunostaining of T-helper cells at the edge of the granuloma (×200). **(D)** CD8 staining showing scarce T-cytotoxic cells within the T-cell rich inflammatory lesions (×200).

**FIGURE 2:**
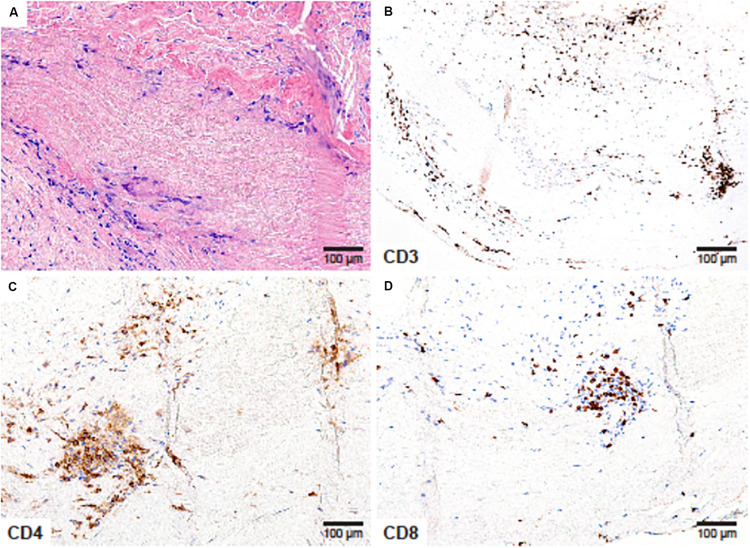
CD4^+^ and CD8^+^ T cells in Takayasu arteritis. Aneurysm of the aortic root and the aortic arch, complicated by aortic regurgitation in a 41-year-old female. **(A)** Necrotizing granulomatous inflammation composed of lymphocytes and multinucleated giant cells surrounding a zone of necrotic medial tissue (H&E ×200); **(B)** CD3^+^ T cells create an inflammatory collar around the zone of necrosis (×200); **(C)** CD4^+^ T-helper cells (×200) and **(D)** CD8^+^ T-cytotoxic cells (×200) comprise the majority of tissue-residing T-cells.

The two most frequent autoimmune diseases affecting the aorta and its branch vessels are giant cell arteritis (GCA) and Takayasu arteritis (TAK), two vasculitides manifesting with aortitis and wall inflammation in the carotid, subclavian, mesenteric and more peripheral arteries (6–8). In both diseases, CD4^+^ T cells and macrophages form granulomatous infiltrates in the vessel wall leading to wall vascularization, loss of medial smooth muscle cells, destruction of elastic membranous lamellae and elastin fibers in the medial layer and growth of lumen-stenosing neointima (6–8) (Figures 1, 2). Damage patterns are similar in GCA and TAK, but the individuals at risk are clearly distinct based on geographic distribution and age at disease onset. Recent advances in non-invasive imaging techniques have demonstrated that the blood vessels targeted by GCA and TAK overlap, giving rise to the ongoing debate whether the two diseases are separate or within the same disease spectrum (9–12). Molecular studies have emphasized that pathogenic events rely on cellular signal transduction pathways that are common in the two diseases, particularly when it comes to upstream pathologic effector functions of CD4^+^ T cells (13–17). However, significant differences mediating disease-relevant processes and more detailed analyses of participating immune cells have supported the proposition that disease-specific activation pathways are potential therapeutic targets (18–20). Here, we will review recent progress in understanding the particular contributions of T cells in disease pathogenesis, how they arrive in the tissue microenvironment of a blood vessel wall, how they function as signal-sending and signal-receiving cells and how their reliance on activating signaling pathways might be exploited therapeutically.

## Giant Cell Arteritis

Earlier studies gave rise to the notion that T cells are clonally expanded within the vascular lesions and that human leukocyte antigen (HLA)-DRB1 alleles are genetic risk factors for GCA (21–24). More recent genetic analyses have confirmed the strong correlation between HLA class I and II molecules and susceptibility to GCA (25, 26). These data provide compelling support to the concept that GCA is a disease in which CD4^+^ T cells react to antigen presented within polymorphic HLA molecules and that antigen recognition and expansion of CD4^+^ T cells are critical disease mechanisms. Here we review different aspects of T cell biology in GCA, with the intent to utilize that knowledge to design more effective therapeutic interventions.

### Effector CD4^+^ T Cells

Multiple studies have shown that Th1 cells that produce interferon (IFN)-γ and Th17 cells that produce IL-17 participate in the vasculitic infiltrates (27–31). An expansion of Th1 and Th17 cells has also been reported for peripheral blood of GCA patients (27, 32). Interferons (IFNs) have a key role in antiviral immunity. IFN-γ, the sole type II IFN, has weaker antiviral effects than type I IFNs, such as IFN-α and IFN-β, but is a potent regulator of various cell types such as endothelial cells, stromal cells, dendritic cells and macrophages (33). IFN-γ vigorously increases major histocompatibility complex expression, increases antigen presentation and amplifies chemokine production, while suppressing cell proliferation (33). IFN-γ is the prototypic macrophage-activating factor that promotes cytokine and chemokine production, phagocytosis, and intracellular killing of microbial pathogens. By releasing IFN-γ, vasculitogenic T cells can effectively activate macrophages and direct their multiple effector functions. In GCA-affected arteries, stimulated macrophages release vascular endothelial growth factor (VEGF), thus fostering neoangiogenesis (34). A spectrum of macrophage functions depends directly on activating signals deriving from IFN-γ-producing CD4^+^ T cells, assigning a key role to these T helper cells in granulomatous vasculitis.

The T cell cytokine IL-17 serves a complimentary role in the disease process. Th17 cells utilize the master transcription factor RAR-related orphan receptor gamma (RORγt) and require IL-23 for lineage differentiation and commitment (35). Besides the classical IFN-γ-supplying Th1 lineage, the so-called “IL-23-IL-17” axis makes critical contributions to autoimmune disease (36). IL-17A has been implicated in barrier and surface protection, functions in neutrophil recruitment and contributes to tissue repair. The “IL-23-IL-17” axis appears to be particularly important in psoriasis and spondyloarthritis (37–39). How IL-17 affects the disease process in GCA is not entirely understood.

Another T cell effector cytokine linked to granulomatous vasculitis is IL-21 (32). IL-21 is predominantly produced by follicular helper T (Tfh) and also by Th17 cells. IL-21 balances helper T cell subsets and induces B cell generation and differentiation into plasma cells, thus enhancing the production of immunoglobulins (40, 41).

IL-9-producing Th9 cells are also enriched in GCA lesions (42). IL-9 is believed to be involved in type 2 inflammation, induces activation of T helper cells and affects the function of various tissue resident cells such as mast cells and epithelial cells in the mucosa. T helper cells make a commitment to the Th9 lineage when stimulated with transforming growth factor β and IL-4 (43). IL-4 is distinctly low in GCA lesions and it is unclear whether Th9 cells are recruited as precursors or fully differentiated. Also, the precise role of IL-9 in GCA lesion remains unknown.

Increased expression of IL-22 in temporal artery biopsies from GCA patients has been reported (44). IL-22 is produced by both innate and adaptive immune cells, including innate lymphoid cells, and natural killer (NK) cells as well as T lymphocytes (Th17 and Th22) (45). IL-22 acts synergistically with TNF-α, IL-1β, and IL-17, and overall has pro-inflammatory effects (46). IL-22 is considered as potential therapeutic target in several autoimmune disease (47–49), but its precise role and drug-ability in GCA requires further investigation.

### CD4^+^ Regulatory T Cells

Regulatory T (Treg) cells, characterized by the expression of the lineage-determining transcription factor FOXP3, have a critical role in the maintenance of immune homeostasis and prevention of autoimmunity (50, 51). Patients with GCA are believed to have insufficient CD4^+^ Treg cell function, eventually resulting in a peripheral tolerance defect (32). Frequencies of CD4^+^FOXP3^+^CD25^*high*^CD127^–^Tregs have been reported to be around 3% in GCA patients, as compared to 4–5% in age-matched controls (32). How this reduction in CD4^+^ Treg cells leads to vasculitis is unresolved. Additional evidence for insufficient Treg cell function came from studies describing enrichment of dysfunctional Treg cells in active GCA patients (52). Specifically, such patients had higher frequencies of IL-17-secreting Tregs, characterized by the expression of an hypofunctional isoform of FOXP3 that lacks exon 2 (52). It has been proposed that tocilizumab, an antibody blocking the IL-6 receptor, may be able to improve the function of Tregs (52). Mechanistic studies, measuring recruitment, stability and suppressive functions of bona fide CD4^+^ Treg cells in the vasculitic lesions are needed to get a better understanding of FOXP3^+^CD4^+^ T cells in GCA.

### CD8^+^ T Cells in GCA

While there is agreement that CD4^+^ T cells are key drivers of both GCA and TAK, CD8^+^ T cells appear to play a disease-specific role. The contribution of effector CD8^+^ T cells to the pathogenesis of GCA is considered to be minor, based on the low number of CD8^+^ T cells in GCA-affected arteries and a marked decrease of circulating CD8^+^ T cells in active GCA patients (53, 54). CD8^+^ T cells in GCA patients have been described to be clonally expanded and to use a restricted T cell repertoire (55, 56). Through which mechanisms effector CD8^+^ T cells may have an impact on pathogenic events in GCA is unresolved. Recent immunohistochemical analyses have confirmed that overall CD8^+^ T cells were lower in GCA-affected vascular lesions compared to Takayasu’s arteritis (57).

**FIGURE 3:**
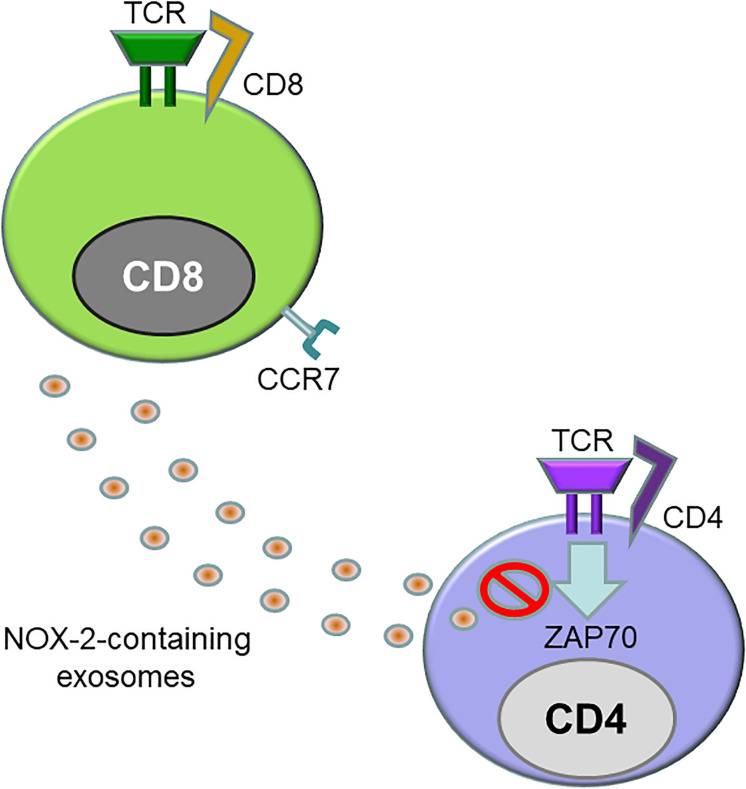
Defective CD8^+^ regulatory T cells in giant cell arteritis. Like CD4^+^ regulatory T cells, CD8^+^ regulatory T cells (CD8^+^ Treg) express the transcription factor FOXP3 and act as a suppressor of immune responses. CD8^+^ CCR7^+^ Tregs inhibit immunity by releasing exosomes that contain the enzyme NADPH oxidase 2 (NOX2). These exosomes are integrated into the membrane of neighboring CD4^+^ T cells, where they disrupt proximal signaling events, including the phosphorylation of ZAP70. CD8^+^ Tregs from patients with GCA are reduced in number and are functionally defective.

One specialized CD8^+^ T cell subset, CD8^+^FOXP3^+^ Treg cells, have been assigned a critical role in the breakdown of the vessel wall immune privilege in GCA (58). Like CD4^+^ Tregs, CD8^+^CD45RA^+^CCR7^+^FOXP3^+^ regulatory T cells have immunosuppressive potential and can be induced *ex vivo* from naïve CD8^+^ T cells by low-affinity T cell receptor signaling combined with IL-15 (59). CD8^+^ Treg cells localize to secondary lymphoid organs in young, healthy individuals, and suppress effector CD4^+^ T cells by inhibiting phosphorylation of ZAP-70, a proximal adaptor molecule in the T cell receptor activation cascade (Figure 3) (58). However, in older individuals and in patients with GCA, CD8^+^ Treg cells are low in numbers and diminished in function. CD8^+^ Treg cells function by releasing NADHP oxidase 2 (NOX2)-containing exosomes, that transfer reactive oxygen species (ROS) into recipient CD4^+^ T cells. Inability to secrete NOX-2-containing exosomes has been identified as the underlying defect of CD8^+^ Treg cells in the old and in the GCA patient (58). Therapeutic targeting of CD8^+^ Tregs, such as increasing functional CD8^+^ Treg numbers or restoring NOX2 production in CD8^+^ Tregs, may control not only GCA but also age-related inflammation or “inflammaging” (58, 60–62).

### T Cell-Macrophage Interactions

Macrophages are immune cells of hematopoietic origin that provide fast immune defense (63). They are equipped to sense and respond to danger signals, usually released from dead and dying cells attacked by infectious microorganisms or other noxious stimuli (64, 65). In GCA, macrophages are unequivocal disease drivers and, together with CD4^+^ T cells, form the pathognomonic granulomatous lesions. They not only produce cytokines (IL-1β, IL-6, and TNF-α) and chemokines (CXCL9, 10, 11, CCL5, and CCL 18) but also contribute to phagocytosis and antigen presentation, and provide co-stimulatory ligands regulating *in situ* T cell activation and survival (66). In the vasculitic lesions, they differentiate into tissue-destructive effector cells by releasing collagenases and matrix metalloproteases (MMP-2, 7, and 9) (67–69). Notably, MMP-9 is almost exclusively produced by CD68^+^ macrophages and controls T cell entry into the vessel wall by digesting the structural integrity of the external basement membrane (68). Blocking MMP-9 efficiently suppressed T cell infiltration into the artery and abrogated the remodeling of the vessel wall, including neointima formation and adventitial neovascularization (68). Essentially, CD4^+^ T cell require MMP-9-releasing macrophages to enter the immune-privileged tissue site and cause vascular inflammation.

While patient-derived macrophages in GCA patients have many features of pro-inflammatory effector cells, their metabolic signature is surprisingly insipid. Expressions of glucose transporters, glycolytic enzymes and transcription factors regulating glycolysis have been described to be indistinguishable from macrophages generated from healthy individuals (66), with healthy and GCA macrophages utilizing glucose as their main substrate. As GCA macrophages enter the tissue microenvironment, they may have access to additional non-glucose energy sources, supporting longevity in the tissue niche. By providing fuel sources adapted to the needs of tissue-invading monocytes and macrophages, the tissue microenvironment attacked by vasculitic immune responses may make a critical contribution to disease pathogenesis.

### T Cell-Dendritic Cell Interactions

Dendritic cells (DCs) are defined as cells with a stellate morphology that can efficiently present antigens on MHC molecules and activate naïve T cells (70). DCs initiate and shape both innate and adaptive immune responses. Considering the reactivity to autoantigen, DCs have long been considered important players in the loss of tolerance leading to autoimmunity, but in recent years their quintessential role in anti-tumor immunity has also been recognized (71, 72). Three-layered human arteries contain a population of DCs, so-called vascular DCs, localized at the adventitia-media border, where they can interact with T cells entering the vessel wall from the adventitial vasa vasorum (73). Vascular DCs sense danger-associated molecular patterns released by pathogens and Toll-like receptor (TLR)-stimulated vascular DCs break self-tolerance and induce T- cell recruitment and activation via co-stimulatory molecule expressions and chemokine release (74, 75). Notably, TLR profiles expressed by vascular DCs are unique for each vascular territory (76).

In addition to co-stimulatory molecules, co-inhibitory molecules are expressed on DCs (77), enabling the DCs to control the initiation, duration and robustness of an immune response. Interactions between programmed cell death protein-1 (PD-1) on activated T cells and its ligand (PD-L1) on DCs have attracted much attention recently since blockade of this axis has anticancer potential (78–81). Our recent work has identified a deficiency of PD-L1 expression on vascular DCs in GCA (82). PD-L1 expression on GCA monocyte-derived DCs was diminished compared to healthy monocyte-derived DCs, even under optimal stimulatory conditions with IFN-γ and LPS. This deficiency leads to overactivation of CD4^+^ T cells, and production of IFN-γ, IL-17, and IL-21 (82). Using a large-vessel vasculitis model, we showed that PD-L1 blockade exacerbates vascular inflammation, promoting infiltration of activated T cells into the arterial wall and wall remodeling with neointima formation and adventitial neovascularization (82). These data provide unequivocal evidence that activated T cells play a central role in vascular remodeling and that anti-tumor therapy with checkpoint inhibitors blocking the PD-1-PD-L1 axis threatens the immune protection of the aorta and its major branches (83). In support of these data, case reports and observational studies have confirmed that patients treated with checkpoint inhibitors are at risk for therapy-induced vasculitis (84, 85). Autoimmunity and tumor immunity emerge as two sides of the same coin. If PD-L1 expression is reduced on DCs, risk for autoimmunity is high, but anti-tumor immunity is effective. In fact, it has been reported that the frequency of malignancy is relatively high in rheumatoid arthritis, Sjogren’s syndrome, inflammatory myositis (86–89); however, the overall risk for cancer in GCA is not increased compared to healthy individuals (90). Understanding why GCA patients have a defect in PD-L1 expression should yield important insights. PD-L1 expression is dependent on glucose uptake and intracellular glycolytic activity (66, 91, 92). Large epidemiological studies demonstrate that body mass index and fasting blood glucose levels were negatively associated with the development of GCA (93, 94), supporting the concept that low glycolytic activity promotes low PD-L1 expression, enabling uncontrolled autoimmunity.

Additional co-inhibitory pathways appear to also be less functional in GCA patients. V-domain immunoglobulin-containing suppressor of T cell activation (VISTA) has been identified as a novel inhibitory receptor expressed on myeloid cells and T cells (95). Expression of VISTA on CD4^+^ T cell from GCA patients has been reported to be decreased, thereby facilitating T cell differentiation toward Th1, Th17, and Tfh (96). Expression of V-set and immunoglobulin domain containing 3 (VSIG-3), a ligand of VISTA (97), has not been explored in GCA lesions.

### Signal Transduction Pathways in GCA CD4^+^ T Cells

#### CD28-AKT-mTORC1 Axis

**FIGURE 4:**
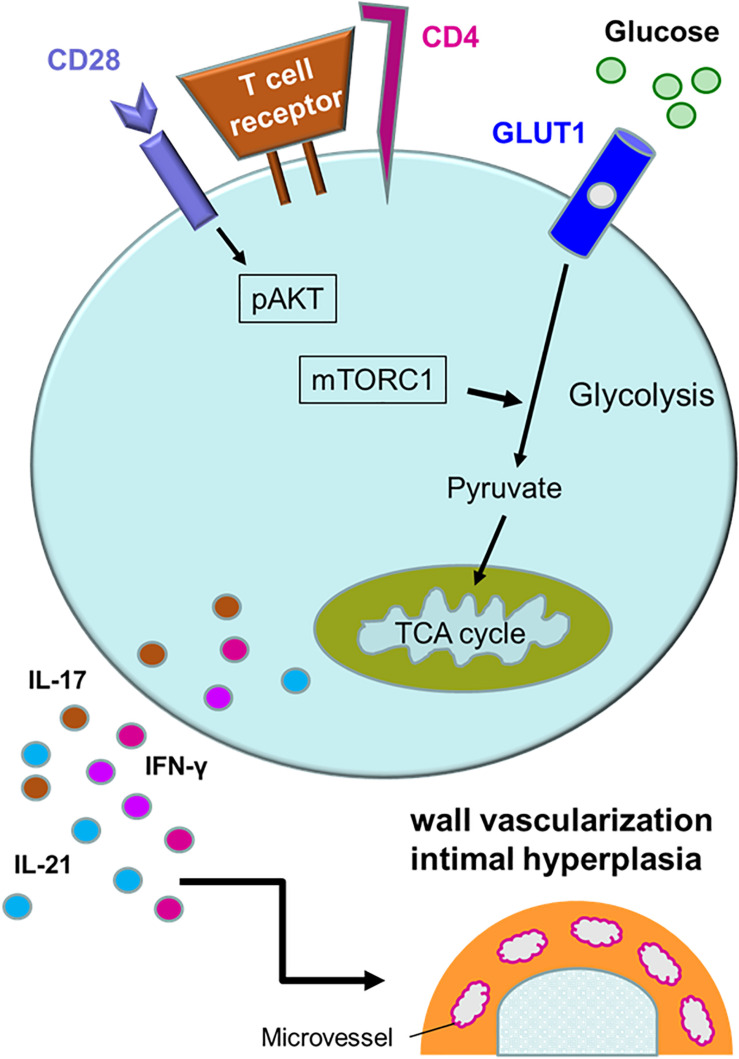
Activation pathways in pathogenic CD4^+^ T cells. Multiple signaling pathways contribute to the activation of pathogenic CD4^+^ T cells in giant cell arteritis. Antigen recognition by the T cell receptor triggers rapid division, differentiation and lineage commitment. CD28 receptor engagement co-stimulates, mediated through AKT phosphorylation and downstream activation of the mechanistic target of rapamycin complex 1 (mTORC1). Additional stimuli derive from metabolic signals. Glucose uptake is controlled through the expression of the glucose transporter 1 (GLUT1). Glycolytic breakdown generates pyruvate, which functions as a critical energy carrier for mitochondria, sustaining ATP production and the release of metabolic intermediates. Antigen-stimulated, metabolically active CD4^+^ T cells differentiate into effector cells secreting IFN-γ, IL-17, and IL-21 and sustain a vessel wall remodeling program resulting in wall vascularization and lumen-occlusive intimal hyperplasia.

Activation of T cells requires at least two signals; one delivered by the T cell receptor complex and one provided by engagement of co-stimulatory receptors, such as CD28 (98, 99). T cell activation initiates a metabolic program required for cell growth, proliferation and differentiation (100). The demand for glucose uptake and glycolysis during T cell activation is known as the Warburg effect (101, 102). CD28 co-stimulation signals the activation of the phosphatidylinositol 3-kinase (PI3K)-AKT axis and maximizes glycolytic flux (103, 104). The mechanistic target of rapamycin (mTOR) is a serine/threonine protein kinase and forms part of mTOR complex 1 (mTORC1) (105). mTORC1 is targeted by the PI3K-AKT axis, and integrates a variety of environmental cues to regulate cell growth and tissue repair (106). mTORC1 promotes glycolysis through upregulation of the hypoxia inducible factor (HIF) 1α (105). mTORC1 is recognized as a signaling hub in multiple pathological conditions, such as cancer, obesity, neurodegeneration and the aging process (107). The PI3K-AKT-mTORC1 pathway is frequently overactivated in various human cancers (108). Sustained activation of mTORC1 is a signature abnormality of CD4^+^ T cells in GCA (Figure 4). In the humanized mouse model of GCA, blockade of CD28 co-stimulation by anti-CD28 antibody was highly effective in dampening vascular inflammation. This therapeutic effect depended on inhibiting mTORC1 activity and constraining glycolytic flux in CD4^+^ T cells (109). Blocking CD28 signaling and preventing mTORC1 activation curtailed mitochondrial respiration and, subsequently, cytokine production (109). Thus, the CD28-AKT-mTORC1 pathway is essential for vasculitic activity and emerges as a promising therapeutic target (13, 15, 109).

#### NOTCH-mTORC1 Axis

**FIGURE 5:**
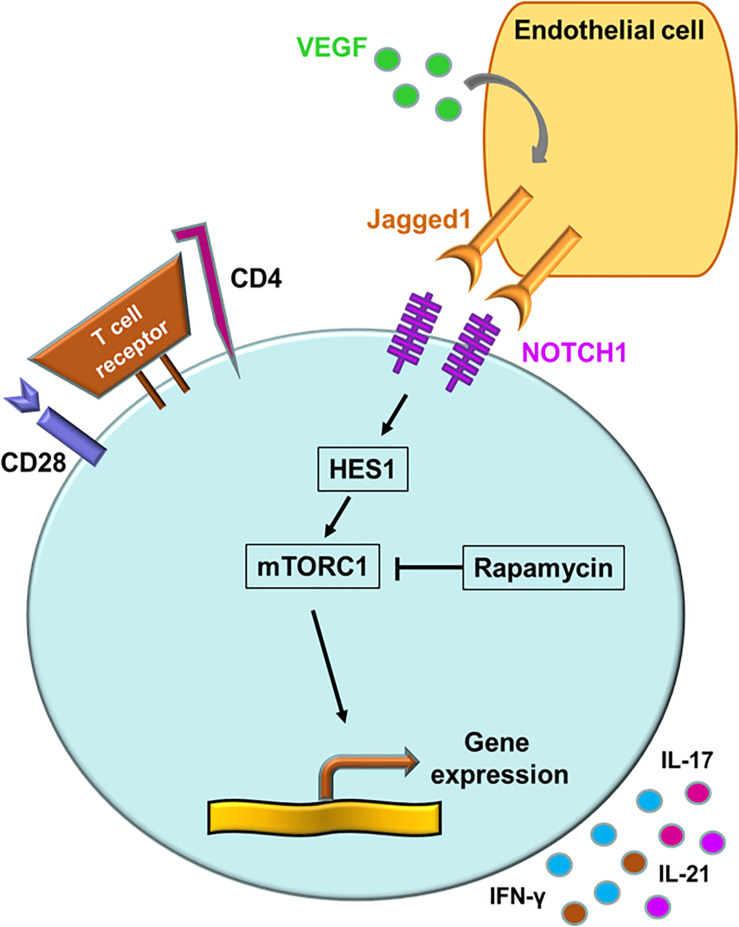
The NOTCH signaling pathway in giant cell arteritis. A signature abnormality of CD4^+^ T cells in GCA is the aberrant expression of the NOTCH1 receptor. NOTCH1^+^ CD4^+^ T cells engage the JAGGED1 ligand on the surface of microvascular endothelial cells. Vascular endothelial growth factor (VEGF) in the circulation functions as the JAGGED1 inducer. Via HES1, the NOTCH signaling pathway activates the mechanistic target of rapamycin complex 1 (mTORC1). Persistent NOTCH signaling promotes T cell proliferation, invasion of CD4^+^ T cells into the vessel wall, upregulation of the metabolic program and differentiation into cytokine-producing effector cells.

“Classical” mTOR inputs are growth factors, nutrients and cellular energy, whereas “non-classical” mTOR inputs include WNT signaling and NOTCH signaling (110). NOTCH signaling primarily regulates cell proliferation, differentiation and cell fate decisions (111). Binding of cell-surface-bound ligands (Delta and JAGGED) to NOTCH receptors on neighboring cells initiates a biochemical cascade that results in of cleavages of the NOTCH receptor. The NOTCH intracellular domain translocates into the nucleus and acts as a transcriptional co-activator that promotes gene expression (110, 111). Aberrant expression of the NOTCH1 receptor is a signature abnormality in CD4^+^ T cells of GCA patients (15). Transcriptomic analysis of biopsy material form GCA patients led to the discovery that the NOTCH1 ligand, JAGGED1, is expressed on microvascular endothelial cells, specifically on the vasa vasorum. VEGF, circulating in high amounts in the blood of GCA patients, was identified as an inducer of JAGGED1 on the endothelial cells (15). Activation of the NOTCH1 pathway resulted in the elevation of the HES1 protein, a potent activator of gene transcription (Figure 5) (15). Taken together, not only the CD28-PI3K-AKT axis but also the JAGGED1-NOTCH1 pathway contribute to high mTORC1 activity, leading to Th1 and Th17 differentiation and to equipping lesional T cells with functionality of disease orchestrators. Recognizing the NOTCH pathway as a driving force in medium and large vessel vasculitis will provide new opportunities for immunomodulation (112).

#### JAK-STAT Pathway

**FIGURE 6:**
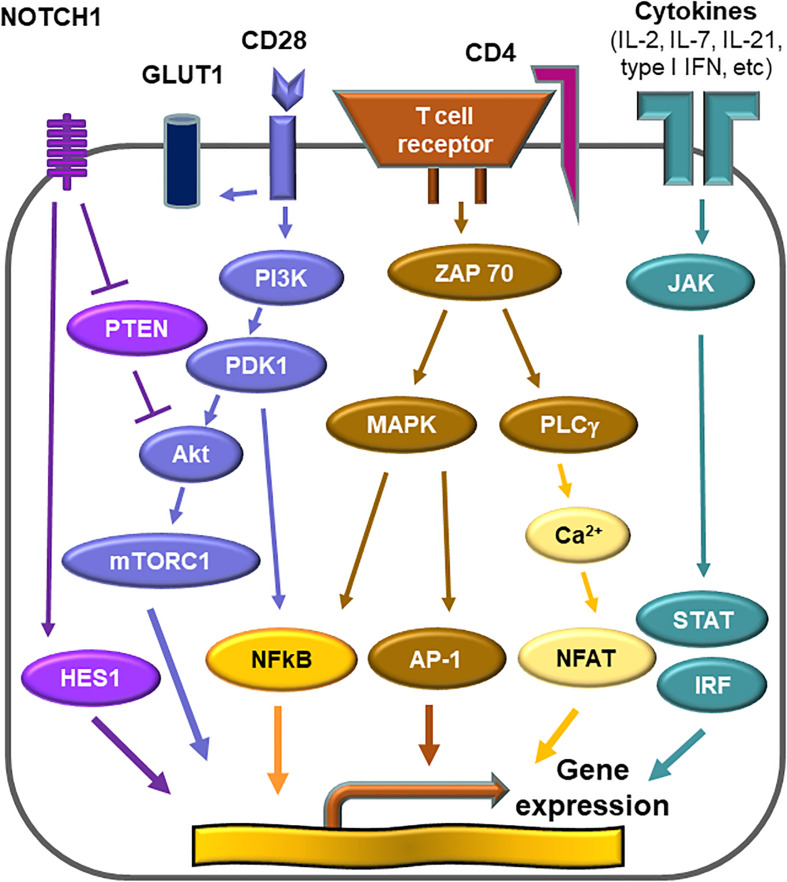
Druggable signaling cascades in vasculitogenic T cells. Numerous signals converge to shape the activation patterns of T cells. In the case of vasculitis-inducing T cells, vasculitogenic antigens trigger the T cell receptor activation cascade. Several co-stimulatory signals adjust signal strength and duration and thus determine differentiation, effector functions and longevity of the T cell. CD28-dependent signaling regulates the metabolic program of vasculitogenic T cells. CD28-mediated signals activate mTORC1, thus determining T cell proliferation and lineage assignment. Persistent NOTCH signaling is a signature abnormality of vasculitogenic CD4^+^ T cells and regulates tissue invasiveness. Cytokines modulating T cell function utilize the JAK-STAT signaling pathway. Disease-relevant cytokine signals derive from IL-2, IL-7, and, possibly, from type 1 interferon.

Cytokines represent soluble factors with essential roles in immune response and employ diverse intracellular pathways. A subset of cytokines that bind type I and type II cytokine receptors utilizes the Janus kinase-signal transducer of activators of transcription (JAK-STAT) pathway (113, 114). They include IL-6, common, chain cytokines (IL-2, 4, 7, 9, 13, and 15), granulocyte colony-stimulating factor (G-CSF), granulocyte macrophage colony-stimulating factor (GM-CSF), IL-10, IFN-α, IFN-β, and IFN-γ (115). Since genome-wide association studies have revealed this pathway as highly relevant to human autoimmunity, pharmacological inhibitors of JAK or JAKinibs have created a new paradigm for the treatment of rheumatic disease (115). In the autoimmune disease rheumatoid arthritis, JAK inhibitors are as effective as biological disease-modifying antirheumatic disease (116, 117), indicating that key disease pathways rely on JAK-STAT signaling. JAK-STAT signaling appears to be exceedingly relevant in vasculitis as well, as recent work in GCA-affected tissue lesions and in patients’ T cells suggests (Figure 6) (16). In human artery-SCID chimera mice, tofacitinib, an inhibitor that primarily targets JAK3 and JAK1, efficiently suppressed vasculitis by restraining the activation of T cells and macrophages (16). Analysis of T cells in the vasculitic infiltrates identified a population of highly proliferative tissue-resident memory T cells, that was dependent on JAK-STAT signaling and sensitive to tofacitinib-mediated inhibition. Such tissue-resident T cells are now emerging as the source of self-renewing T cells, that maintain the granulomatous formations in the vessel wall. In a clinical study, re-biopsy of patients after 3, 6, 9, and 12 months of corticosteroid therapy demonstrated that the vascular inflammatory lesions were unexpectedly stable and tissue-occupying T cells persisted in the majority of patients for more than 1 year (118). These “tissue-resident memory T cells” are now recognizes as a specialized T cell subset, that can adapt to the tissue environment and retain the capacity to locally replenish the infiltrate. These T cells express CD103 and CD69 as a phenotypic surface marker (119–121). In a *trans-*engraftment model, in which human arteries with vasculitis lesions are engrafted into “empty” immunodeficient mice, the survival strategy of human T cells can be analyzed. In these *trans-*engrafted arteries, tissue-resident memory T cells expand autonomously and their expansion is druggable with JAKinib (16). Tofacitinib was sufficient to diminish cell proliferation and cytokine production from activated CD4^+^ T cells (16), demonstrating that JAK-STAT signaling contributes to disease-relevant processes in GCA.

#### Type I IFN Signature

Interferons (IFN) are cytokines that have antiviral, antiproliferative, and immunomodulatory effects (122). Inborn errors or impaired function of IFN-mediated immunity confer predisposition to viral and mycobacterial infection (123). There are two main classes of IFN: IFN-γ is the only type II IFN, and type I IFNs include IFN-α, IFN-β, and others that bind a common cell-surface receptor (122). Type I IFNs activate the JAK-STAT signaling pathway to induce expression of interferon-stimulated genes, called “interferon signature” (Figure 6) (33). Interferons also induce an “interferon epigenomic signature” by activating latent enhancers and chromatin (33). The type I IFN signature is upregulated in several autoimmune diseases, identifying this cellular activation pathway as a prime candidate for immunosuppressive therapy (124, 125). Transcriptome analysis of biopsy material from patients with GCA has demonstrated robust induction of type I and II IFN signatures (16), indicating that both type I and type II IFNs are abundant within the vasculitic lesions. The source of type I IFNs in GCA has not been defined, providing an opportunity to identify key cellular drivers. In the autoimmune disease systemic lupus erythematosus, plasmacytoid DCs are the main source of type I IFN (126, 127). There is no evidence, to date, that plasmacytoid DC are present in the inflamed vessel wall. The effect that IFNs have on T cells is complex, involving direct and indirect interference in T cell functionality (128). Type I IFN receptors are broadly expressed in many tissues and by many cell types, both innate as well as adaptive immune cells are regulated by this cytokine family. Receptors for type II IFN are mostly encountered on granulocytes, monocytes and macrophages, Type II IFN is a product mainly of T cells and NK cells, placing the T cell-monocyte/macrophage axis at center stage in GCA.

## Takayasu’s Arteritis

Unlike GCA, that has the highest incidence in elderly women of Northern European descent (8, 129), Takayasu’s arteritis (TAK) is more prevalent in young Asian women (130–132). HLA-B52 is recognized as a susceptibility locus in TAK (20, 133–135). A subset of TAK patients have pre-existent or co-existent inflammatory bowel disease, connecting this vasculitis to systemic immune abnormalities (136, 137). HLA-B52 is detected in a high rate of patients with TAK complicated with ulcerative colitis (138). The vascular lesions of TAK resemble those encountered in GCA and are composed of highly activated T cells and macrophages, arranged in granulomatous formations (6). In the past decade, TAK and GCA have often been considered to represent a spectrum of one disease, supported by similarities in target blood vessel patterning and histopathologic findings (9–12). However, genome-wide associated studies and immunophenotyping of immune cells in the peripheral blood and in the vascular lesions have revealed substantial differences (18–20, 57). Particularly, immune cells other than CD4^+^ T cells and macrophages have shown to be critical in the pathogenesis of TAK (19, 20, 139, 140). Here, we review the current knowledge implicating CD4^+^ T cells, macrophages, CD8^+^ T cells, natural killer (NK) cells as critical players in the disease process leading to TAK. New data demonstrating the production of autoantibodies against endothelial cells in TAK patients shed new light on pathogenic adaptive immunity (141). Special emphasis has been placed on cellular signaling pathways that are active in TAK and GCA, with the goal to define common and disease-specific pathogenic mechanisms.

### CD4^+^ Effector T Cell

Giant cell arteritis and Takayasu arteritis share the predominance of Th1 cells and the participation of Th17 cells in the inflammatory process (141). IFN-γ and IL-17 are markedly increased in the peripheral blood and in the affected aortic tissue (141). The entire signaling pathways mediating the induction of Th1 cells appears to be upregulated. *IL-12B* is well established as a susceptibility gene in TAK (134), and plasma IL-12 levels are elevated (142), biasing T cell differentiation toward the Th1 lineage (143). IL-23 is also increased in the serum of patients with TAK (141), and IL-23 promotes IL-17 production by CD4^+^ T cells (144). In essence, the cytokine environment enables the emergence of Th1 and Th17 cells, promoting immune responses associated with granulomatous inflammation.

Differential responses to glucocorticoid therapy have been reported for GCA and TAK patients. In GCA, Th17 cells are susceptible, while Th1 cells are resistant to glucocorticoid therapy; whereas steroid treatment is able to suppress Th1 cytokines, but left Th17 cytokines unaffected in TAK (27, 141). The reason for this strikingly different responsiveness remains unclear, but these data strongly support the hypothesis that the cytokine environment may ultimately be very different in these two disorders.

A shared feature of CD4^+^ T cells in both GCA and TAK is the strong upregulation of mTORC1 activity (13, 14). mTOR is a signaling hub in T cell fate decisions and mTORC1 activation biases T cell differentiation toward the Th1 and Th17 lineage. Strong evidence for mTORC1’s role as a key decision maker comes from studies using rapamycin to guide T cell differentiation. The mTORC1 blocker was able to prevent differentiation into Th1 and Th17 cells (13, 14). The cause of persistent mTORC1 activation in GCA and TAK T cells is not understood but may be an independent factor in directing disease-relevant immunity. Upregulation of the mTORC1 pathway has also been reported for endothelial cells and vascular smooth muscle cells in the aorta (13, 145), suggesting that the mTORC1 pathway is universally activated in TAK.

In a recent study, a group of French scientists explored whether IFN signatures are activated in isolated CD4^+^ and CD8^+^ T cells from patients with active TAK (17). Transcriptome analysis demonstrated that 248 genes were dysregulated in CD4^+^ T cells and 432 genes in CD8^+^ T cells. Pathway enrichment analysis identified type I and type II IFN signatures and cytokine/chemokine signaling as highly enriched in both CD4^+^ and CD8^+^ T cells from TAK patients. Further analysis pointed toward active signaling in the STAT5 pathway, suggesting that the patients’ T cells might be exposed to elevated levels of the T cell growth factor IL-2. Treatment of two TAK patients with a JAK-STAT inhibitor resulted in a measurable decreased of Th1 and Th17 cell frequencies (17), supporting the premise that T cells activation in TAK is ultimately controlled by cytokines and growths factors utilizing the JAK-STAT pathway. This conclusion is supported by a series of case reports showing the efficacy of JAK inhibitors in refractory TAK patients (146, 147). These studies suggest a potential role of JAK-STAT pathway blockade as a promising approach to dampen disease activity in TAK (148).

### CD4^+^ Regulatory T Cells

Common to most autoimmune diseases, Treg cell dysfunction has been proposed to underlie the chronic immune stimulation in TAK. Recent work has shown a decrease in the function of peripheral blood CD4^+^ Treg cells in TAK patients (149). Plasticity of CD4^+^ Tregs acquiring effector cell function has been described (150, 151), and the authors proposed that Th2-like transformed Tregs that secret IL-4 and IL-13 contribute to the development of TAK (149). Th2 cytokines are rarely encountered in GCA (152), and the appearance of this class of cytokines may indeed be a distinguishing features between TAK and GCA. Selective therapeutic manipulation of CD4^+^ Tregs in autoimmunity has long been an objective (51) and the above-mentioned study reported that the blockade of the JAK-STAT pathway can restore CD4^+^ Treg cells and increase the ratio of CD4^+^Treg/CD4^+^ effector T cells (17). Correction of the aberrantly activated JAK-STAT pathway may, therefore, be effective in modifying several pathogenic domains in TAK. However, this form of treatment in TAK raises a number of safety concerns, all of which need to be addressed before new therapies can be introduced into standard management; including the age of the patients, the chronicity of disease and the risk of immunosuppression during the pandemic spread of new viral agents, promotion of neoplasia, etc.

### CD8^+^ T Cells

Detailed analyses of the inflammatory burden in TAK have demonstrated CD8^+^ T cells accounting for approximately 15% of the wall-infiltrating cells in TAK-affected aortas (153). CD8^+^ T cells are able to release perforin directly onto the surface of aortic vascular cells, thus causing direct tissue damage (153). Immunophenotypic studies of peripheral blood immune cells using multiparametric fluorescent techniques have provided evidence that the numbers of total CD8^+^ T cells and CD8^+^ effector T cells are both higher in TAK than in GCA (19). Notably, memory CD8^+^ T cells remained high even during clinical remission and the number of total CD8^+^ T cells was correlated with TAK, but not GCA relapse (19). In TAK patients, CD8^+^ T cells are increased not only in the circulation but also in the vessel wall infiltrates (57). Immunohistochemical analysis has shown that, compared with GCA-affected arteries, vascular surgical specimens in TAK had increased proportions of CD8^+^ T cells and the CD4/CD8 ratio differentiated the two vasculitides (57). A recent GWAS study also supported the critical role of CD8^+^ T cells in the pathogenesis of TAK (20). Taken together, these results indicate that GCA and TAK have critical differences in immune cell composition in the peripheral blood and within the vasculitic lesions.

CD4^+^ T cells and CD8^+^ T cells in TAK patients share sustained mTORC1 activity (14, 154, 155). Upstream signals that drive activation of the mTORC1 pathway have not yet been defined and our understanding of the functional consequences for protective and pathogenic immunity in these patients is limited. Continuous activation of central cellular signaling pathways appears to be an overall theme in patients affected by TAK. Besides chronic mTORC1 activation, T cells from TAK patients have ongoing activation of the JAK-STAT pathway, inducing type I and type II IFN gene expression signatures (17). Comparative studies of CD4^+^ and CD8^+^ T cells, which are responsible for fundamentally different effector pathways, may shed light on the state of chronic stimulation of the adaptive immune system in this form of vasculitis.

### Natural Killer (NK) Cells

Seko et al., were the first to report that NK cells have a prominent position in the vascular injury leading to TAK (153). CD16^+^ NK cells account for 20% of the immune cells in the aortic wall infiltrates (153). Like CD8^+^ T cells, NK cells were positive for perforin immunostaining, providing strong support for a role in inducing cellular damage. Further support for the critical contribution of NK cells has come from the GWAS study by Terao et al., defining NK cells as the most promising target in the pathophysiology of TAK (20). NK cells are regulated by accessory molecules, such as the major histocompatibility complex (MHC) class I chain-related gene (MIC) family, and the leukocyte immunoglobulin-like receptor (LILR) family (139). Overexpression of MIC-related A (MICA) and its receptor natural killer group 2 member D (NKG2D) in aortic tissue from TAK cases has been reported (156). Expression of MHC and MICA on aortic vascular cells allows NK cells to recognize them through the NKG2D receptor and, in turn, attack them (139). LILR family members (LILR A1 to A6 and LILR B1 to B5) are widely expressed on hematopoietic cells and mediate activation as well as inhibition of immune cell function (157–159). Among them, *LILRA3* was identified as a novel susceptibility loci in the TAK GWAS study (20). How precisely LILRA3 regulates immune response in TAK remains largely unknown, but it has been proposed that LILRA3 may bind to the major TAK susceptibility molecule HLA-B52 (20, 139). So far, NK-directed therapies are unavailable but TAK may become the signature disease to tap into novel opportunities of immune modulation and preventing immune cell-dependent cellular injury.

### Autoantibodies Against Endothelial Cells (ECs)

A recent report on anti-endothelial cell autoantibodies has raised the question of the potential role that B cells participate in TAK disease pathogenesis. B cells are infrequent in the vasculitic infiltrates, which are typically cell admixtures of T cells and macrophages. However, B cells may play a critical role in the breakdown of immune tolerance that precedes the invasion of the vessel wall by inflammatory cells. Anti-endothelial cell antibodies (AECA) are a heterogenous group of autoantibodies against ECs (160). AECA have been detected across the spectrum of systemic vasculitides, ranging from small-vessel vasculitides to medium- and large-vessel vasculitides (161). Classically, AECA bind to ECs and induce apoptosis through direct complement-dependent cytotoxicity or through indirect antibody-dependent cytotoxicity (161), identifying antibodies as regulators of EC survival. In TAK, IgG deposits have been reported in the intima (162) and autoantibodies recognizing ECs have been evaluated as disease activity markers (163, 164). As expected, identification and quantification of AECA varies based on the technique used, e.g., indirect immunofluorescence, enzyme-linked immunosorbent assay, fluorescence-activated cell sorting, and immunoblot assays (160). Accordingly, measurement of AECA has not found its way into routine diagnostic schemes. This may change as a recent publication has defined autoantigens recognized by AECA. Mutoh et al., have identified two autoantigens expressed on ECs by applying an elegant serological identification system based on a retroviral expression system and flow cytometry (165). In this system, a cDNA library of ECs was retrovirally transfected into a rat myeloma cell line and AECA-positive clones were sorted by flow cytometry (165, 166). This yielded endothelial protein C receptor (EPCR) and scavenger receptor class B type 1 (SR-BI) as bona fide autoantigens (165). In a cohort study, the authors found that approximately 1 in 3 patients with TAK produce autoantibodies against either antigen. Remarkably, both molecules that are recognized by autoantibodies negatively regulate endothelial cell function, undermining the protective role of the endothelial layer (165). The authors propose that the autoantibodies disrupt the barrier function of ECs, opening the intimal surface to immune cell infiltration. Autoantibodies recognizing antigen specifically expressed by ECs appears to accompany chronic inflammatory disease (167, 168), but, interestingly, the autoantigens display a disease-specific pattern. Understanding the timing of events such as the emergence of AECA may be informative in assigning AECA-specific pathogenic determinants.

## Conclusion

The inaccessibility of the body’s major arteries to tissue sampling have complicated the diagnosis and pathogenic understanding of autoimmunity in blood vessels. With the advent of non-invasive imaging techniques, vasculitis of the aorta and its major branch vessels can now be assessed, classified, and monitored. Comprehensive analysis using DNA, RNA, proteins, cell surface markers, transcription factors, and signaling pathways have greatly contributed to our understanding of the pathophysiology of large vessel vasculitis. A major insight has been that LVV has two principle disease components, the systemic inflammatory response, and the granulomatous vasculitis in the vessel wall. Traditionally, it has been assumed that patients with GCA and TAK develop autoimmunity against vascular antigens, which induces granulomatous vasculitis and, as a spill over, systemic inflammation. Much improved conceptualization of autoimmunity is beginning to question the validity of this traditional disease concept.

Patients with GCA and TAK are born with genetic risk factors that render them susceptible to a disease that will present clinically 2–6 decades later. Genes within and outside of the HLA complex have been identified as risk determinants, but a consistent theme of the association studies is the connection of genetic risk factors with immune cell function. With solid evidence that GCA and TAK are essentially immune-mediated diseases, multiple immune cell types are now recognized as critical disease players, including CD4^+^ and CD8^+^ T cells, monocytes and macrophages, NK cells and autoantibody-producing B cells. Emerging data indicate that CD8^+^ T cells may be more important in TAK than in GCA, opening opportunities to implicate different immune cells in different aspects of pathogenesis (Table 1). Pathogenic studies in TAK have been complicated by multiple hurdles, such as access to diseased tissue, the lack of reliable animal models and the low disease prevalence. Therefore, data on disease mechanisms in TAK have remained less robust. In both, GCA and TAK, immune cell-mediated injury to vascular cells may lie upstream of the chronic granulomatous reaction typifying these autoimmune diseases.

**TABLE 1:** CD4^+^ and CD8^+^ T cells in giant cell arteritis and in Takayasu arteritis.

		**Giant cell arteritis**	**Takayasu arteritis**
CD4^+^	Contribution to the disease	Key driver	Key driver
	Dominant helper T cell subset	Th1, Th17, Th21, Th9	Th1, Th17, Th21, Th9
	Dominant effector molecules	IFN-γ, IL-17, IL-9, IL-21, IL-22	IFN-γ, IL-17, IL-9, IL-21
	CD4^+^ Treg	Dysfunctional	Decreased
	mTORC1	Highly activated	Highly activated
	NOTCH pathway	Highly activated	??
	JAK-STAT pathway	Highly activated	Highly activated
	Type I Interferon signaling	Activated	Activated
CD8^+^	Contribution to the disease	Minimal	Substantial
	Dominant effector molecule	??	Perforin
	CD8^+^ Treg	Decreased and dysfunctional	??
	mTORC1	??	Highly activated
	JAK-STAT pathway	??	Highly activated
	Type I Interferon signature	??	Activated

As in other autoimmune diseases there is now recognition that pathogenic events leading to GCA and TAK may involve multiple disease components, that are not always coordinated and that require specialized therapeutic interventions. Specifically, the extravascular and the vascular component of LVV seem to follow different trajectories, rely on different mechanisms, and respond differently to current treatments. Extravascular GCA and TAK are characterized by intense systemic inflammation and are measured by elevation of acute phase reactants, such as C-reactive protein and erythrocyte sedimentation rate. They are simple to measure in the peripheral blood and are easy to suppress with corticosteroids or by blocking IL-6 signaling (169, 170). Much more challenging is the vascular component of TAK and GCA; a persistent, refractory granulomatous inflammation positioned in the vessel wall (69, 171). Vascular GCA and TAK are difficult to treat; 50% of patients have persistent vasculitis despite intense therapy for 1 year (118). The resistance to standard immunosuppression is corroborated by recent reports of ongoing disease activity in patients treated with anti-IL-6 therapy (172–176). Underlying molecular mechanisms are those of sustained activation of innate and adaptive immune cells through a plethora of signaling pathways. Most significant are the enduring activation of the mTOR pathway, the NOTCH signaling pathway and the JAK-STAT pathway (Table 1 and Figure 6). In combination, these essential cellular signaling pathways drive lasting immune responses in a tissue site that is intolerant to damage. The unparalleled effectiveness of corticosteroids in treating large vessel vasculitis may well reflect their imprecision in suppressing cellular activation pathways.

## Author Contributions

RW, JG, and CW wrote the manuscript. GB contributed the tissue images. DL participated in the concept development. All authors contributed to the article and approved the submitted version.

## Conflict of Interest

The authors declare that the research was conducted in the absence of any commercial or financial relationships that could be construed as a potential conflict of interest.
